# Assessing the accuracy and quality of artificial intelligence (AI) chatbot-generated responses in making patient-specific drug-therapy and healthcare-related decisions

**DOI:** 10.1186/s12911-024-02824-5

**Published:** 2024-12-24

**Authors:** Meron W. Shiferaw, Taylor Zheng, Abigail Winter, Leigh Ann Mike, Lingtak-Neander Chan

**Affiliations:** School of Pharmacy, University of Washington, 1959 NE Pacific Street, Box 357630, Seattle, WA 98195 USA

**Keywords:** ChatGPT, Artificial intelligence, Drug therapy, Pharmacotherapy, Healthcare decisions

## Abstract

**Background:**

Interactive artificial intelligence tools such as ChatGPT have gained popularity, yet little is known about their reliability as a reference tool for healthcare-related information for healthcare providers and trainees. The objective of this study was to assess the consistency, quality, and accuracy of the responses generated by ChatGPT on healthcare-related inquiries.

**Methods:**

A total of 18 open-ended questions including six questions in three defined clinical areas (2 each to address “what”, “why”, and “how”, respectively) were submitted to ChatGPT v3.5 based on real-world usage experience. The experiment was conducted in duplicate using 2 computers. Five investigators independently ranked each response using a 4-point scale to rate the quality of the bot’s responses. The Delphi method was used to compare each investigator’s score with the goal of reaching at least 80% consistency. The accuracy of the responses was checked using established professional references and resources. When the responses were in question, the bot was asked to provide reference material used for the investigators to determine the accuracy and quality. The investigators determined the consistency, accuracy, and quality by establishing a consensus.

**Results:**

The speech pattern and length of the responses were consistent within the same user but different between users. Occasionally, ChatGPT provided 2 completely different responses to the same question. Overall, ChatGPT provided more accurate responses (8 out of 12) to the “what” questions with less reliable performance to the “why” and “how” questions. We identified errors in calculation, unit of measurement, and misuse of protocols by ChatGPT. Some of these errors could result in clinical decisions leading to harm. We also identified citations and references shown by ChatGPT that did not exist in the literature.

**Conclusions:**

ChatGPT is not ready to take on the coaching role for either healthcare learners or healthcare professionals. The lack of consistency in the responses to the same question is problematic for both learners and decision-makers. The intrinsic assumptions made by the chatbot could lead to erroneous clinical decisions. The unreliability in providing valid references is a serious flaw in using ChatGPT to drive clinical decision making.

## Introduction

Chat Generative Pre-trained Transformer (ChatGPT) is a language processing tool driven by artificial intelligent (AI) chatbot launched by OpenAI in November 2022. It belongs to the family of large language models fine-tuned with supervised and reinforcement learning techniques. It is trained on a vast corpus of text data and can answer various questions, engage in conversations, and generate text on multiple topics using predictive text [1, 2]. It has gained popularity in its ability to write essays and code based on simple prompts and is being utilized by students and professionals in many settings globally. According to one internet-based survey, 43% of professionals have used AI tools, including ChatGPT, for work-related tasks [3–5]. 

As AI technology advances and becomes more accessible, it serves as another source of information for healthcare-related needs for learners, patients and healthcare workers [6]. There are several concerns about healthcare information provided by ChatGPT (and other generative AI tools), including whether the information is accurate and current; how to effectively use the bot to obtain accurate information; and whether clinical decisions are soundly made [7, 8]. In a study aimed to evaluate the performance of ChatGPT on answering questions within the scope of the United States Medical Licensing Examination using multiple choice questions, ChatGPT achieved an accuracy rate between 44-64.4%.^9^ In another study, ChatGPT was asked questions using multi-part cases that are administered to pre-clerkship medical students by a medical school in the US, and the responses generated by ChatGPT achieved a passing score of 69% in only 12 of the 28 responses [9]. Similarly, ChatGPT did not pass the 2023 Taiwanese Pharmacist Licensing Examination, achieving a correct response rate between 54.5 and 67.6%.^11^ These findings raise concerns about the appropriateness and safety of using ChatGPT as a primary reference source or study guide on healthcare-related topics.

While it is interesting to see the performance of chatbots in taking exams, we are particularly concerned about the quality of information provided by ChatGPT through real-world uses and the associated risks. Based on our experience and direct observations, some trainees and healthcare providers have been using ChatGPT as their primary tool for learning and studying; looking up healthcare information; and consulting ChatGPT to assist with clinical decision making. We have also observed professional students using ChatGPT as a reference source to complete open-book quizzes and examinations. Because AI is an evolving field, there is very limited experience or published data in assessing quality of healthcare information provided by ChatGPT, either as a study tool or in guiding clinical decision making. Our study was intended to explore this important issue to provide guidance on the use and misuse of this technology at this time.

It is essential to understand the limitations and strengths of AI technology, such as ChatGPT, to maximize its potential and recognize its limitations as a source of healthcare information. In this study, we investigated whether ChatGPT could be safely utilized in healthcare-related inquiries. Specifically, we aimed to: (i) determine the consistency of responses provided by ChatGPT to the questions posed; and (ii) assess the quality of responses by ChatGPT to prompts that asked “what,” “why,” or “how” questions with focus on drug therapy and general disease management.

## Methods

We submitted a total of 18 questions to ChatGPT (free online version by OpenAI, GPT3.5 series) to assess the quality of the responses, including six questions (2 questions each to address what, why, and how, respectively) in three defined clinical areas. The “what” questions addressed simple fact-based inquiries (e.g., What anticonvulsants require renal dose adjustments? What are the commercially available dosage forms and strengths for warfarin). The “why” questions expected the chatbot to provide explanation of a specific therapeutic action or plan (e.g., why do we use ACE inhibitors or angiotensin blockers in patients with proteinuric chronic kidney disease? Why would a patient with diabetes not be started on metformin at diagnosis). The “how” questions involved asking the chatbot to provide a plan based on a scenario (e.g., How do I educate a 16-year-old patient with type 1 diabetes and celiac disease on how to treat low blood sugar?) We used the following focused clinical areas: anticoagulation therapy in atrial fibrillation, diabetes management, and chronic kidney disease (CKD). These areas were chosen because of their broad applicability to general practice and relevance to drug therapy, which provided a stronger basis to evaluate the fact-based responses by ChatGPT. All 5 investigators independently developed 6 questions in these 3 areas. These topic areas and questions were chosen because they are within the expertise of the investigators. These questions were also similar to those asked by learners and clinicians based on real-world in-person group discussion sessions, case teaching conferences, or consultation cases through experiential learning. The questions were phrased to simulate the language early-stage students in healthcare fields would utilize or use with the chatbot. The investigators then reviewed all the questions together to reach a consensus for the final 18 questions to be presented to ChatGPT (Table 1).

**Table 1:** Final 18 questions entered to ChatGPT for consultation responses

Category	Prompt Type	Questions
Atrial fibrillation	What	1. What are the commercially available dosage forms and strengths for warfarin? 2. What are the GI bleeding rates associated with for each of the DOACs in older adults?
	Why	3. Why don’t we start anticoagulation therapies in all patients diagnosed with atrial fibrillation? 4. Why would we choose warfarin over a DOAC for atrial fibrillation?
	How	5. How do we initiate amiodarone for a 65-year-old patient with atrial fibrillation currently stable on warfarin? 6. A 62-year-old man has been taking warfarin 7.5 mg daily for atrial fibrillation. His INR today is 1.7. How should I adjust his warfarin dose?
CKD	What	1. What anticonvulsants require renal dose adjustment? 2. What disease states predispose a patient to chronic kidney disease?
	Why	3. Why do we use ACE inhibitors or ARBs in patients with proteinuric CKD? 4. Why should we avoid NSAIDs in patients with CKD?
	How	5. How do we manage vancomycin therapy in a 61-year-old man who weighs 150 lbs with a serum creatinine of 2.3? 6. How do we determine the IV iron dose in a 57-year-old woman with CKD, hemoglobin of 9.2, and ferritin of 35 requiring hemodialysis?
Diabetes	What	1. What is the hemoglobin A1c goal for patients with diabetes? 2. What is the difference between type 1 and type 2 diabetes?
	Why	3. Why would we choose an SGLT2 inhibitor over a GLP-1 agonist for diabetes? 4. Why would a patient with diabetes not be started on metformin at diagnosis?
	How	5. A frail 62-year-old woman with type 2 diabetes and hypertension has an A1C of 6.4%. Her current A1C goal is less than 7%. She has experienced two falls in the last 6 months. How should I adjust her A1C goal? 6. How do I educate a 16-year-old patient with type 1 diabetes and celiac disease on how to treat low blood sugar?

The experiment was conducted in duplicate. The final 18 questions were entered into the chat box in ChatGPT independently by 2 co-investigators (TZ and MS) on 2 laptop computers, and two different internet browsers (Chrome for TZ and Safari for MS). This allowed us to compare the consistency of the responses. The responses were transcribed into an electronic spreadsheet to allow for side-by-side comparison. We used a 4-point scale to score the quality of the bot’s responses, with a score of 0 for completely wrong or inaccurate responses and 3 for accurate response that is consistent with current practice standards (Table 2). Each of the 5 investigators independently scored all 36 responses to determine accuracy and consistency. The Delphi method was used to compare the score for each of the responses with the goal of reaching at least 80% consistency (i.e., 4 of 5 investigators assigned the same score). In case of agreement < 80% during the Delphi vote (i.e., more than 1 person disagreed with the score), the bot’s response was thoroughly discussed and rescored until at least 4 investigators reached the same score.

**Table 2:** Scoring rubric for ChatGPT responses

Overall Assessment	Score	Basis in assigning the score
Response is wrong/inaccurate	0	• Is the response accurate? • If the response is wrong → what part of it is wrong?
Response is incomplete or unclear	1	• Is the response consistent with the question asked?
Response is mostly correct with some missing or outdated information	2	• Example: partial response but not specific to the question • The response includes making multiple assumptions • General response but outdated
Response is accurate and consistent with practice standards	3	• Exactly what is expected, or as stated in credible sources.

Each chatbot-generated response was fact-checked and verified using credible professional resources such as the latest practice guidelines, current textbooks, or established professional electronic references (Table 3). The scores for each response generated from each of the 2 laptop computers were compared for consistency. The scores for the questions categorized as what, why, or how were expressed as mean ± standard deviation for comparison purposes.

**Table 3:** References used to verify response from ChatGPT

PubMed	National Institute of Diabetes and Digestive and kidney diseases website (https://www.niddk.nih.gov/health-information/diabetes)
UpToDate	CDC
Micromedex	Manufacturer’s Package Insert
American Diabetes Association (ADA) Diabetes Care Vol 46, Supplement 1. (https://diabetesjournals.org/care/issue/46/Supplement_1)	Facts & Comparisons

## Results

### Consistency of responses

In general, the speech, format, and length of the responses were consistent within the same user but were different between the 2 users. The consistency of response between users did not follow a specific speech pattern and changed based on the type of question. The responses to the “what” questions were more likely to be consistent in the anticoagulation therapy and CKD categories, but inconsistent in the diabetes category. On several occasions, ChatGPT provided completely different responses to the same question posed by 2 different users. For example, to the question “what is the hemoglobin A1c goal for patients with diabetes?” ChatGPT responded with “7.5 to 8%” for one user but “6.5%” for the other user. For reference purpose, the A1c goal is less than 7% for most adult patients with diabetes according to the current recommendation by the American Diabetes Association. Similarly, to the question “what anticonvulsants require renal dose adjustment?”, oxcarbazepine and tiagabine were listed in the response for one user but were not included in the response for the other user. Overall, there were discrepancies in the responses generated for the same question between users.

### Quality and accuracy of responses

Regarding the quality and accuracy of the responses provided, ChatGPT generated the most accurate responses to the “what” questions with 8 of the 12 questions receiving a score of 3 (indicating an accurate response consistent with current practice standards). Of note, responses to 2 of the 10 “what” questions also received a score of zero due to inaccuracy. Both questions answered incorrectly related to renal dose adjustment for anticonvulsants, in which ChatGPT suggested to renally dose-adjust two agents (carbamazepine and phenytoin) that, based on available information and the pharmacokinetics of the drugs, do not require adjustments. These discrepancies may lead to erroneous calculations of drug doses leading to either suboptimal therapy or toxicity. The number of responses receiving a score of zero were 3 and 2 for the “why” and “how” categories, respectively. Using the ratio between the number of responses with scores of 3 versus 0 within each of the 3 question categories as an indicator of the performance of ChatGPT on accuracy, the calculated ratios were 4, 1.67, and 2 for “what”, “why”, “how” responses, respectively. A score of 0 was assigned by all 5 investigators to one “what,” question, one “how” question, and two “why” questions. Using the total score as the performance indicator by adding up the score received per question within each of the 3 categories, they were 27, 22, and 12, for “what”, “why”, and “how”, respectively (Tables 4 and 5).

**Table 4:** Total score of responses from ChatGPT. There are 6 questions from each of the three categories. The range of the scores is between 0 (wrong answer) to 3 (fully accurate result). The maximal achievable point for each category is 36 (i.e., a score of 3 in all 12 responses)

Question Category	Total Points
How	12
What	27
Why	22

**Table 5:** Summary of ChatGPT performance on responses based on best (score = 3) and worst (score = 0) responses

	Total Number of Accurate Responses (score = 3)			Total number of Inaccurate Responses (score = 0)		
	what	why	how	what	why	how
User 1	5	2	1	1	2	3
User 2	3	3	1	1	1	1

### Computational performance

There were multiple instances where ChatGPT showed computational errors. In response to the question, “How do we manage vancomycin therapy in a 61-year-old man who weighs 150 lbs with a serum creatinine of 2.3?”, ChatGPT provided the general dosing recommendation of vancomycin in mg/kg, followed by the recommended regimen for this case patient. However, we identified 2 major areas of concern. First, ChatGPT did not confirm the unit of measurement for serum creatinine concentration and assumed that the value was expressed as mg/dL to perform the dose calculation. Second, in one of the responses to this question, ChatGPT showed the Cockcroft-Gault equation in estimating creatinine clearance, and then provided recommended vancomycin regimen based on the creatinine clearance. However, when we manually double-checked the calculation, the weight calculated by ChatGPT was slightly different (68.18 vs. 68.04 kg; a 0.2% difference). The most surprising finding was obtaining a different value of creatinine clearance when we performed the calculation manually using a standard calculator based on the exact equations and numbers generated by ChatGPT. The creatinine clearance between the calculator-generated and ChatGPT-generated values differed by 12% (32.6 mL/min vs. 36.3 mL/min).

### Major errors or misinformation in qualitative responses

In addition to the computational errors, we identified several serious errors in the qualitative responses. When given the prompt “How do we initiate amiodarone for a 65-year-old patient with atrial fibrillation currently stable on warfarin?”, the ChatGPT response only offered the typical maintenance doses for amiodarone but failed to mention the necessity of a loading dose for either user. In response to the prompt “A 62-year-old man has been taking warfarin 7.5 mg daily for atrial fibrillation. His INR today is 1.7. How should I adjust his warfarin dose?”, the response from one of the users suggested decreasing the daily warfarin dose to 6.4 mg instead of increasing the dose, as would be clinically indicated. In response to the prompt “Why should we avoid NSAIDs in patients with CKD?”, the response from one user failed to mention any bleeding risk associated with NSAID use. Finally, in response to the prompt “How do I educate a 16-year-old patient with type 1 diabetes and celiac disease on how to treat low blood sugar?”, for both users ChatGPT provided generic recommendations to treat hypoglycemia such as glucose tablets, juice, or candy; however, it failed to take the patient’s celiac disease into consideration in either response and did not mention concerns about gluten-containing products.

### References and citations to support responses

For responses generated by ChatGPT that we considered to be inaccurate or unclear, we asked ChatGPT to provide reference sources. Some of the references or citations provided by ChatGPT could not be confirmed or were erroneous. This included citations that could not be confirmed using PubMed or the Digital Object Identifier (DOI) addresses on the internet, or citations showing completely different papers when we visited the journal using the cited year, volume, and pages. In response to the prompt “Why would we choose an SGLT2 inhibitor over a GLP-1 agonist for diabetes?”, ChatGPT suggested that SLGT2 inhibitors “may lead to modest weight gain”. When asked to provide a reference, ChatGPT summarized one of the studies by responding with “This study found that patients treated with canagliflozin experience weight gain compared to those on a placebo.” Upon further review, the published study clearly stated that the patients in the SGLT2 inhibitor arm experienced weight loss rather than weight gain. There were also instances where the references were outdated. For example, in responding to the prompt, “A 62-year-old man has been taking warfarin 7.5 mg daily for atrial fibrillation. His international normalized ratio (INR) today is 1.7. How should I adjust his warfarin dose?” ChatGPT recommended *decreasing* the dose of warfarin by 10–15%, however, this INR result in practice would warrant a warfarin dose increase. When asked to provide a reference to the nomogram used in the answer, ChatGPT provided the 2008 American College of Chest Physicians (ACCP) Chest Guidelines as the source (though having incorrectly interpreted the information provided within), despite updated ACCP Chest Guidelines having been published in 2021 [10]. Additional examples of inaccurate and/or inconsistent response, and AI hallucinations are summarized in Table 6.

**Table 6:** Highlight of examples of inaccurate responses, inconsistent responses, and examples of AI hallucinations

**Example 1- Inaccurate recommendation that can cause serious harm**	
Prompt: A 62-year-old man has been taking warfarin 7.5 mg daily for atrial fibrillation.	
His INR today is 1.7. How should I adjust his warfarin dose?	
Excerpt of chatbot response:	Assessment of response:
“….*Use a nomogram or an online calculator to determine the patient’s new dose of warfarin. * *For example*,* the following nomogram can be used*: *INR 1.6–1.9: Decrease weekly dose by 10–15%* *INR 2.0–3.0: No change* *Based on this nomogram*,* the patient’s dose should be decreased by 10–15%. Calculate the new weekly dose of warfarin: 52.5 mg/week x 0.85 = 44.6 mg/week (using a 15% reduction) Determine the new daily dose of warfarin: 44.6 mg/week ÷ 7 days = 6.4 mg/day. Therefore*,* the new warfarin dose for this patient should be 6.4 mg daily*.”	• Reducing warfarin dose in this case can lead to a thromboembolic event; • A 6.4 mg daily dose is not a viable or practical option based on the availability dosage form. • When asked to provide a reference to the nomogram used in the answer, ChatGPT provided the 2008 American College of Chest Physicians (ACCP) Chest Guidelines
**Example 2- Inconsistent results/responses**,** with in accurate information**	
Prompt: Why would we choose an SGLT2 inhibitor over a GLP-1 agonist for diabetes?	
Excerpt of chatbot responses:	Assessment of responses:
In one response (user 1) – “….*if the patient is overweight or obese*,* SGLT2 inhibitors may be preferred as they have a greater impact on weight loss*” In another response (user 2)- “….*GLP-1 agonists are associated with weight loss*,* whereas SGLT2 inhibitors are weight-neutral or may even lead to modest weight gain*”	• The responses to the exact same question generated by 2 computers are completely contradictory. • When user 1 asked ChatGPT for the sources or citations that support weight loss with SGLT2 inhibitors, the following reference was provided: Rosenstock J, et al. Effects of Dapagliflozin, an SGLT2 Inhibitor, on HbA1c, Body Weight, and Hypoglycemia Risk in Patients With Type 2 Diabetes Inadequately Controlled on Pioglitazone Monotherapy. Diabetes Care 2012;35(7):1473–1478. • However, when we reviewed the article, it was very clear that compared with placebo, dapagliflozin was not associated with weight loss in this 48-week trial. Weight gain was observed at week 48.
**Example 3- Incomplete responses with AI Hallucination (reference cannot be identified)**	
Prompt:	
What are the GI bleeding rates associated with each of the DOACs in older adults?	
Chatbot responses:	Assessment of responses:
In one response (use 1), specific bleeding rates were mentioned. In the response for use 2, there were no specific bleeding rates mentioned at all. Instead, the ChatGPT provided generic, non-specific statements regarding the risk of GI bleeding.	• We asked ChatGPT to provide 3–5 references to support the summary and conclusion. One of the citations was Dentali F, Gianni M, Riva N, et al. Direct oral anticoagulants and gastrointestinal bleeding in elderly adults: a prospective cohort study. Aging Clin Exp Res 2020;32(5):791–799. • However, we were unable to identify the above paper through a PubMed search and google search. A complete review of cited medical journal for the papers published 2020 also failed to identify such publication. An independent PubMed search using a combination of authors’ names also failed to identify such publication.
**Example 4- Consistently incorrect information**	
Prompt: What anticonvulsants require renal dose adjustments?	
Chatbot response:	Assessment of responses:
Phenytoin and carbamazepine were both listed as drugs requiring renal dose adjustment in the responses to both users 1 and 2.	• Based on the pharmacokinetics, neither drug requires routine dose adjustment based on renal function • Neither the manufacturer’s package inserts nor the standard drug information references recommend routine renal dose adjustment

## Discussion

AI and machine learning have been incorporated in clinical medicine for over two decades. Interpretation of medical images such as cutaneous lesions and retinal photographs, histopathology analysis, electrocardiogram, and white-cell differential counts are examples of machine-learning aided tasks already in place in clinical medicine [11–13]. A chatbot such as ChatGPT differs from existing data learning machines in that it uses AI and natural-language processing to understand questions and automate responses to them, simulating human conversation. It can be more engaging to humans and potentially serve as a scribe, a coach in assisting clinical documentation, or to help guide clinical decision making [13]. Our study shows that ChatGPT has potential but is not ready to take on the coaching role for either healthcare learners or healthcare professionals. The lack of consistency in the responses to the same question is problematic for both learners and decision-makers. The assumptions made in decision-making without verification, such as unit of measurement, could lead to erroneous clinical decisions. The unreliability in providing valid references is a serious flaw in driving clinical decisions.

Previous studies tested ChatGPT on multiple choice-based questions and demonstrated adequate but not outstanding performance [14, 15]. Our study is more consistent with real-world use in that we asked ChatGPT open-ended questions and qualitatively analyzed the responses from the bot. To our knowledge, there are very few studies assessing ChatGPT on qualitative responses [9, 16]. The results showed that ChatGPT’s performance is mediocre at best. One of the investigators suggested that the chat bot “should not be relied upon without expert human supervision” [16]. Our study has confirmed these conclusions.

Similar to our research, recent investigations assessing chatbot-generated responses to medical queries by physicians and pharmacists also showed that the chatbot’s showed promises but had significant limitations. Goodman et al. showed that ChatGPT provided more accurate response to general medical queries that were simple and straight forward but less accurate and incomplete with more complex questions [17]. Huang et al. showed that ChatGPT performed optimally in providing non-specific drug counseling information but poorly when responses required incorporating patient-specific situations, and in recognizing adverse drug reactions [18]. Our study is unique in that we compared the performance of ChatGPT based on the type of qualitative questions submitted, namely the “what”, “why”, and “how” questions. The focus of this study was on patient management which complements other previously published research focusing on diagnosis [19–21]. Our results showed that ChatGPT performed the best in fact-based questions, especially when prompted with “what”. The performance of ChatGPT was inferior for questions requiring explanation or providing recommendation through processing/analyzing information. Categorically speaking, ChatGPT had the lowest performance in the “how” questions, where 8 of the 12 responses received a score of 0 or 1.

One of the biggest concerns from our experience is AI hallucinations with ChatGPT. AI hallucinations is a term used in the tech industry to describe fabrications and definitive statements on uncertain history, fact, and information by AI [22]. AI hallucinations with ChatGPT have been associated with historical documents, reports, names and dates that do not exist, or even historical events that never happened. AI hallucinations in science have also been reported, including ChatGPT presented reference papers that do not exist [23]. A recent study aimed to investigate the frequency of AI hallucinations in research proposals entirely drafted by ChatGPT showed that 28 out of 178 or 15.7% of references generated by ChatGPT neither turned up on Google search nor had an existing DOI [24]. Our study has confirmed these observations. Fabrication of information in healthcare inquiries can lead to dire consequences, such as making wrong clinical decisions that causes harm to patients. We believe this is the most dangerous aspect of non-discriminative use of ChatGPT at this point. The cause of AI hallucinations is unclear but likely multifactorial. Proposed contributors include exposure to biased data, insufficient training data, and intrinsic flaws in the machine language learning model [25, 26]. Until there is a reliable approach in preventing AI hallucinations, chatbot generated clinical information must be verified using credible resources. Clinical recommendations and decisions should not be made by chatbots without careful review and approval by humans with relevant expertise in the topics. Recently, AI hallucinations led to an open investigation by the US Federal Trade Commission into OpenAI to determine if the inaccuracies of ChatGPT has caused harm to consumers [27]. Overall, these findings suggest that at the present time, ChatGPT cannot replace established clinical references such as UpToDate, Micromedex, Facts and Comparisons, or a manufacturer’s package insert, especially relating to drug information. More importantly, since the data sources used for training ChatGPT in health-related information is unclear, learners and clinicians should be advised that ChatGPT can only be used to facilitate information gathering but should not be used alone as an “expert” advisor. All content generated by ChatGPT must be fully reviewed and verified.

The strengths of our study include using open-ended questions. The questions were worded to simulate the language early-stage students in healthcare field would utilize or use with the chatbot in real-life. The duplicate experimental design allows us to assess the consistency of the response. We purposedly constructed questions that addressed “what”, “why” and “how” to compare the performance of of the chatbot. And we interacted with the Chatbot to obtain references which allowed us to further evaluate AI hallucinations. Our study has several weaknesses. We have focused our experiments on only three defined disease areas. Since AI is a rapidly evolving field, the performance of the updated versions of ChatGPT or other chatbot may be different. Although newer versions of ChatGPTs are now available, ChatGPT v3.5 is still the most accessible version since it is free, making our experience and results highly relevant. The differences in responses between users suggest that the variance of chatbot responses may be even more significant that our observation. This confirms that AI chatbot technology should be used with caution.

In conclusion, we do not recommend using ChatGPT as the sole reference source for clinical information or as a learning coach, due to lack of test-retest reliability, inter-user variability of responses, and the uncertain trustworthiness based on unreliable sources. The risk of harm posed by incorrect information and assessment can result in serious negative consequences. We continue to recommend using gold standard professional references to approach patient-specific problems. If ChatGPT is used, we urge that all information provided by ChatGPT at the present time be verified using established clinical or professional references. We conclude that ChatGPT cannot be safely used in healthcare, given the level of inaccuracy shown in this study.

## Data Availability

The datasets used and/or analysed during the current study are available from the corresponding author on reasonable request.
